# Comparative analysis of single-cell transcriptomics in human and Zebrafish oocytes

**DOI:** 10.1186/s12864-020-06860-z

**Published:** 2020-07-08

**Authors:** Handan Can, Sree K. Chanumolu, Elena Gonzalez-Muñoz, Sukumal Prukudom, Hasan H. Otu, Jose B. Cibelli

**Affiliations:** 1 Department of Electrical and Computer Engineering, University of Nebraska-Lincoln, Lincoln, NE 68588 USA; 2 LARCEL, Andalusian Laboratory of Cell Reprogramming (LARCel), Andalusian Center for Nanomedicine and Biotechnology-BIONAND, 29590 Málaga, Spain; 3 Department of Cell Biology, Genetics and Physiology, University of Málaga and; Networking Research Center on Bioengineering, Biomaterials and Nanomedicine, (CIBER-BBNE), 29071 Málaga, Spain; 4 Department of Anatomy, Faculty of Veterinary Medicine, Kasetsart University, Bangkok, 10900 Thailand; 5 Departments of Animal Science and Large Animal Clinical Sciences, Michigan State University, East Lansing, MI 48824 USA

**Keywords:** Zebrafish, Oocyte, Orthology, RNA-seq, Transcriptome

## Abstract

**Background:**

Zebrafish is a popular model organism, which is widely used in developmental biology research. Despite its general use, the direct comparison of the zebrafish and human oocyte transcriptomes has not been well studied. It is significant to see if the similarity observed between the two organisms at the gene sequence level is also observed at the expression level in key cell types such as the oocyte.

**Results:**

We performed single-cell RNA-seq of the zebrafish oocyte and compared it with two studies that have performed single-cell RNA-seq of the human oocyte. We carried out a comparative analysis of genes expressed in the oocyte and genes highly expressed in the oocyte across the three studies. Overall, we found high consistency between the human studies and high concordance in expression for the orthologous genes in the two organisms. According to the Ensembl database, about 60% of the human protein coding genes are orthologous to the zebrafish genes. Our results showed that a higher percentage of the genes that are highly expressed in both organisms show orthology compared to the lower expressed genes. Systems biology analysis of the genes highly expressed in the three studies showed significant overlap of the enriched pathways and GO terms. Moreover, orthologous genes that are commonly overexpressed in both organisms were involved in biological mechanisms that are functionally essential to the oocyte.

**Conclusions:**

Orthologous genes are concurrently highly expressed in the oocytes of the two organisms and these genes belong to similar functional categories. Our results provide evidence that zebrafish could serve as a valid model organism to study the oocyte with direct implications in human.

## Background

The implementation of zebrafish (*Danio rerio*) as an animal model to study human disease is growing at an unprecedented pace [1]. The applications span a wide range and include models for neurological disorders, aging, cancer, behavior, pharmacology, and toxicology, among others [2–7].

The fact that its embryo is transparent, placed zebrafish as one of the main vertebrate models to study developmental processes [8]. It has been shown that cellular and molecular events leading to and governing gastrulation, the formation of the primitive streak, and organogenesis in zebrafish show great parallels with mammals [9–11]. However, less is known about the differences and similarities between the female gametes.

Here, we sought to compare the transcriptome profile of the single matured human and unfertilized zebrafish oocytes at the time of ovulation. Our study shows that despite the significant evolutionary distance between humans and zebrafish, the mature female gametes of both species have significant similarities in gene expression.

## Results

### Gene expression by type

**Fig. 1:**
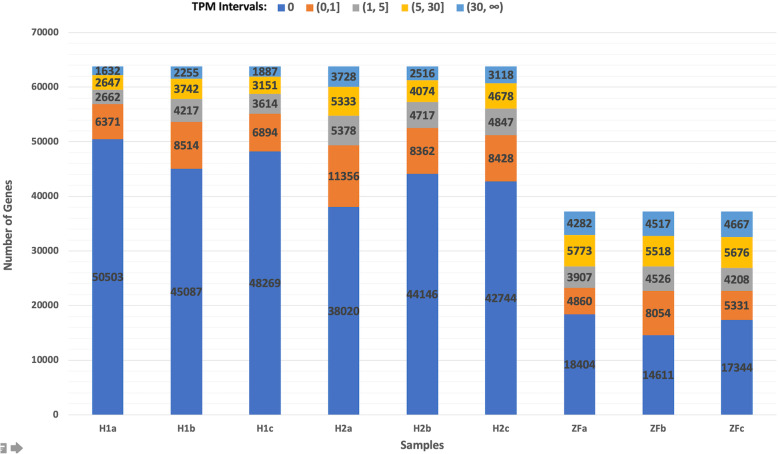
Transcripts per million (TPM) distribution for the nine samples used in our analysis: TPM values are divided into five intervals for each sample and the number of genes in each interval are shown. Biological replicates are indicated with lower case letters, a,b,c. Sample order follows the two human datasets (H1 and H2) followed by our zebrafish dataset (ZF)

Our data analysis involve three single-cell RNA-seq datasets for the oocyte, each with three samples: zebrafish data generated by our group (ZF), human dataset 1 (H1) [12] and human dataset 2 (H2) [13]. In Fig. 1, we show the transcripts per million (TPM) distribution for each of the nine samples used in our analysis. As expected, most of the genes showed very low or no expression; on average 75, 65, and 45% of the genes had zero TPM, and 87, 80, and 61% of the genes had less than one TPM in the H1, H2, and ZF datasets, respectively. The smaller percentage of genes with little-to-no expression in zebrafish was due to the lower number of identified pseudogenes in the zebrafish genome, which tend to have low read assignments. In the supplementary data (Supplementary file 1), we break down the TPM distribution for each of the 9 samples (3 samples each coming from the 3 datasets) based on the 46 and 30 gene types described in human and zebrafish, respectively.

About 88% of the gene abundance comes from protein-coding genes in human (90% for the H1 and 86% for the H2 datasets), whereas in zebrafish, this ratio is around 79%. In human, most of the noncoding gene abundance comes from mitochondrial ribosomal RNAs (Mt-rRNAs) and long intervening noncoding RNAs (lincRNAs). In zebrafish, the lincRNA abundance is less significant with most of the noncoding gene abundance coming from Mt-rRNAs and rRNAs (Supplementary file 1).

### Orthologous gene expression

**Fig. 2:**
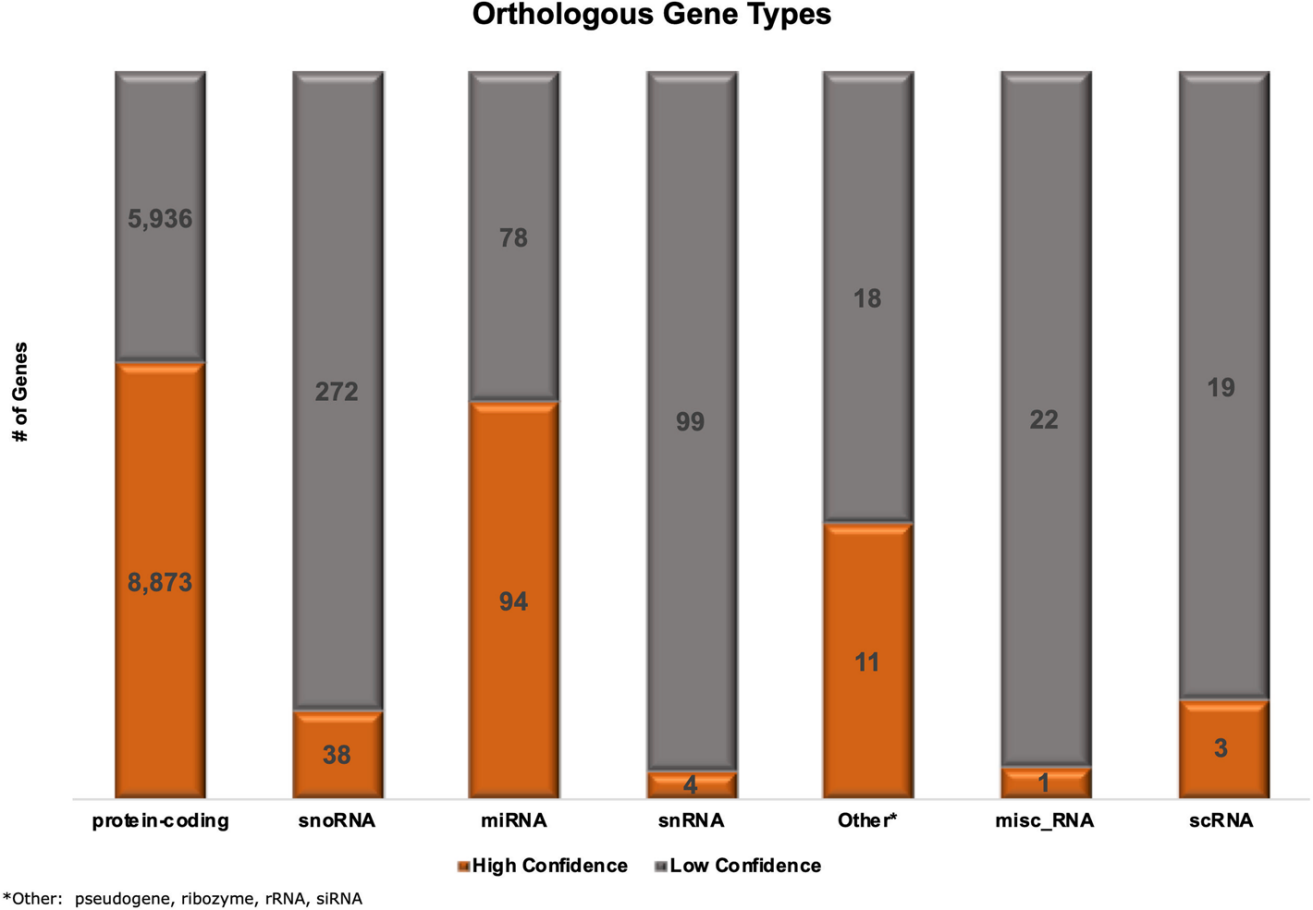
Gene types that form an orthologous pair between human and zebrafish

There are 18,388 orthologous gene pairs defined between the two organisms in the Ensembl database. These gene pairs involve many-to-many mappings, i.e., one human gene may be orthologous to more than one zebrafish gene; and there may be more than one human gene orthologous to the same zebrafish gene. The 18,388 orthologous gene pairs involve 13,963 human genes and 16,546 zebrafish genes. The Ensembl database further groups the orthologous gene pairs as high-confidence and low-confidence orthology. There are 9809 high-confidence orthologous gene pairs between the two organisms, and this mapping involves 9020 human genes and 9495 zebrafish genes. In Fig. 2, we summarize the types of genes involved in the orthology mapping and their confidence levels. Approximately 60% of the human protein-coding genes have an orthologous zebrafish gene.

**Fig. 3:**
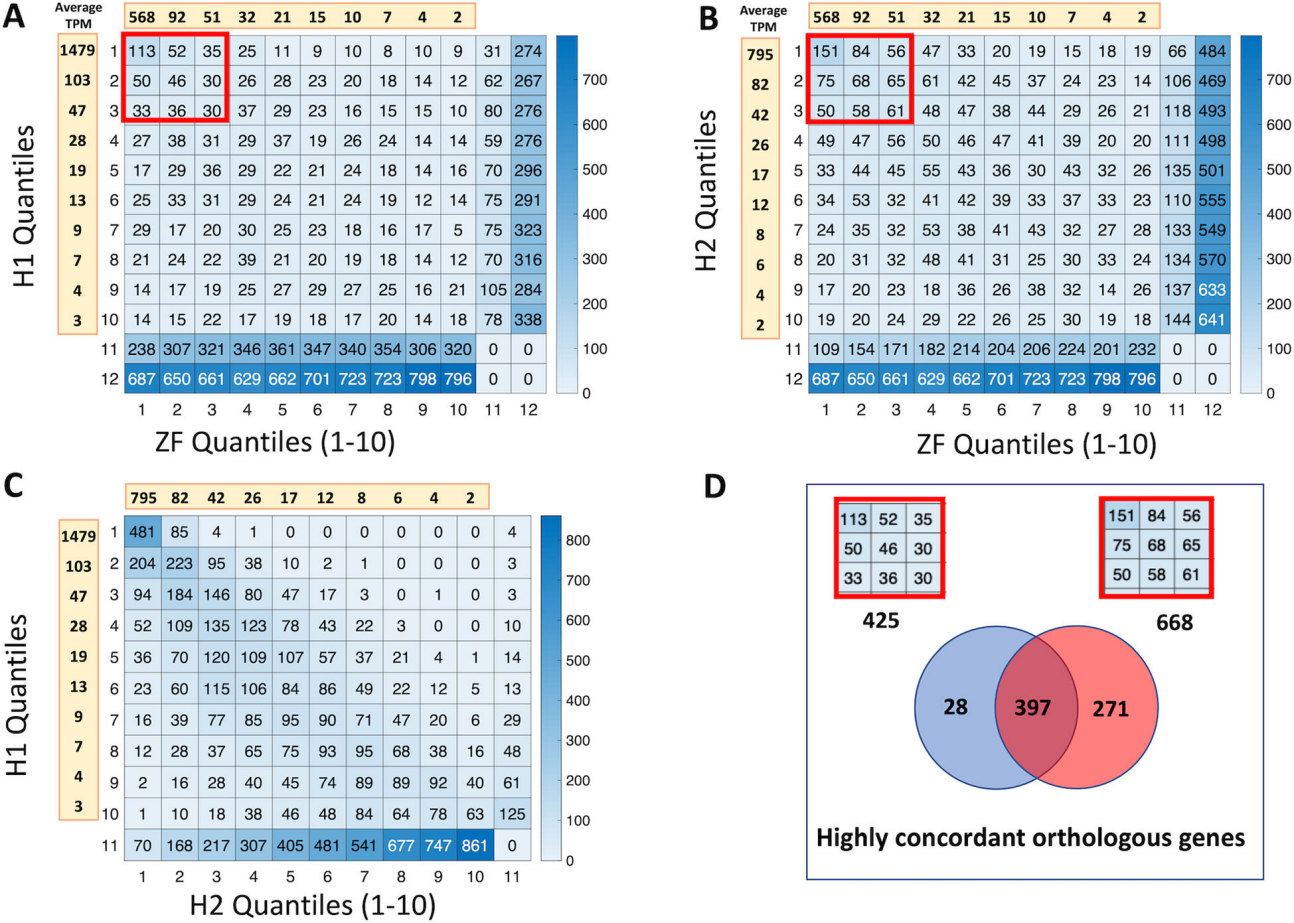
Quantile mapping between pairs of data sets: (**a**) H1 vs. ZF, (**b**) H2 vs. ZF, and (**c**) H1 vs. H2. For each mapping, a heatmap shows the number of common genes in each quantile. For across organism mappings (**a** and **b**), Row 11: genes that are expressed in zebrafish, have a high-confident orthologue in human, but are not expressed in human; Row 12: genes that are expressed in zebrafish but do not have a high-confidence orthologue in human; Column 11: genes that are expressed in human, have a high-confident orthologue in zebrafish, but are not expressed in zebrafish; Column 12: genes that are expressed in human but do not have a high-confidence orthologue in zebrafish. For H1-H2 mapping, Row/Column 11 identify the genes that are expressed in only one of the datasets. For each quantile, we also show the average TPM value shown in data value bars with a yellow background. In (**d**), we summarize the overlap between the top 30% of highly expressed (the 3 × 3 top-left corner of the quantile mappings in **a** and **b**) genes that are high-confidence orthologs across the two organisms for the H1 and H2 datasets

In order to identify the expression of orthologous genes between the two organisms, we first identified genes that are “expressed” in a dataset as the genes that have a TPM value higher than one in all three biological replicates used in the dataset. This resulted in 5753, 9917, and 12,383 genes expressed in the H1, H2, and ZF datasets, respectively. There were 5443 genes common in the expressed gene lists for the two human datasets showing ~ 95% overlap between them. We then divided the expressed genes in each dataset into 10 quantiles, i.e., the first quantile consists of the top 10% of the most highly expressed genes in the dataset, etc. We compared the genes in each quantile across pairs of datasets, which we termed “quantile mapping.” In Fig. 3, we show the mapping results for each of the three pairwise comparisons; and in the supplementary data (Supplementary file 2), we show the genes in each of the cells shown in Fig. 3 with corresponding annotations, sample-level signal values, and z-scores. During the quantile mapping between the human and zebrafish datasets, we considered only the high-confidence orthologous genes retaining the cases that render many-to-many mappings as described above.

The quantile mapping between H1 and H2 shows that the 95% similarity between the two gene sets also follows the same TPM distribution as the large mapping numbers are observed along a diagonal (Fig. 3c). Therefore, not only do we see a high overlap among the genes expressed in the two human datasets, but these genes are also expressed at approximately the same relative levels in the two oocyte sets, underscoring the quality of the datasets. Our results for across organism mappings suggest that more than 50% of the genes expressed in the human oocyte have an orthologue that is also expressed in the zebrafish oocyte: 3174 for H1 and 5057 for H2 (data not shown). When only the high-confidence orthologs are considered, these numbers drop down to 2314 for H1 and 3657 for H2, accounting for ~ 40% of the genes expressed in the human oocytes (Fig. 3a, b). However, more importantly, these genes are concentrated on the top-left region of the quantile mapping heatmap. In other words, a higher percentage of the genes that are highly expressed in both organisms show high-confidence orthology compared to the lower expressed genes. For example, when H1 is compared to ZF, the 2314 high-confidence orthologous genes are distributed into 10 × 10 = 100 quantile mapping cells (Fig. 3a). Therefore, on average, we would expect ~ 23 genes to be in each cell for a random distribution. However, the very top-left cell, which represents the genes that are in the top 10% in both datasets and are high-confidence orthologs, for example, has 113 genes. This is a very significant occurrence (*p* < 10^− 21^, Fisher’s exact test) showing that high-confidence orthologous genes are concurrently highly expressed in the oocytes of the two organisms.

A similar observation holds for H2. Out of the 3657 genes expressed in H2 with a high-confidence ortholog in zebrafish that is also expressed in ZF, 151 are in the top 10% in the two organisms (*p* < 10^− 25^). This significance of occurrence does not just hold for the top-left cell in the quantile mapping but for the top-left region, as well. For example, if we focus on the top-left 3 × 3 corner of the quantile mapping results, i.e., high-confidence orthologous genes that are expressed in the top 30% in both of the organisms, we see 425 genes mapped for H1 (*p* < 10^− 12^) and 668 genes mapped for H2 (*p* < 10^− 14^). On the other hand, out of the genes that are expressed in the human oocyte and have a high-confidence ortholog in zebrafish (2812 for H1 and 4524 for H2; Fig. 3a, b), only about one-fifth are not expressed in the zebrafish oocyte (575 for H1 and 997 for H2; Fig. 3a, b). The genes that are expressed in the human oocyte and have a high-confidence ortholog in zebrafish comprise the total number of “unique” human genes in the quantile mapping that span Rows 1–10 and Columns 1–11. Among these, the unique human genes in Column 11 are the ones not expressed in zebrafish (Fig. 3a, b Supplementary file 2).

### Highly concordant orthologous genes

The 425 and 668 genes that are high-confidence orthologs between the two organisms and appeared in the top 30% of the expression bracket for ZF as well as for H1 and H2 datasets, respectively, showed ~ 93% overlap, or 397 genes (Fig. 3d, Supplementary file 3). Based on the average TPM of the 9 samples, in Table 1 we show the top 25 of the 397 genes that we call “highly concordant orthologous genes.” In this table, we show only the top representative of a gene group, e.g., “mitochondrially encoded cytochrome c oxidase,” or “ribosomal protein.”

**Table 1:** Top 25 genes that are orthologous between human and zebrafish and expressed in the top 30% of all three data sets. Average TPM was calculated using all nine samples. For a gene family, e.g., “ribosomal proteins,” only the top representative is listed. The complete list of genes can be found in Supplementary file 3

Rank	GeneID (ENSG00000+)	Symbol	Description	Average TPM
1	198712	MT-CO2	Mitochondrially encoded cytochrome c oxidase	16,481
3	198886	MT-ND4	Mitochondrially encoded NADH	9839
7	198899	MT-ATP6	mitochondrially encoded ATP synthase	8180
9	130816	DNMT1	DNA methyltransferase 1	5089
10	132646	PCNA	Proliferating cell nuclear antigen	4380
11	173207	CKS1B	CDC28 protein kinase regulatory subunit	4273
13	138326	RPS24	Ribosomal protein	2701
15	182004	SNRPE	Small nuclear ribonucleoprotein polypeptide E	2497
19	137707	BTG4	BTG anti-proliferation factor 4	1994
21	120533	ENY2	Transcription and export complex 2 subunit	1796
23	113387	SUB1	SUB1 homolog, transcriptional regulator	1663
24	113558	SKP1	S-phase kinase associated protein 1	1656
25	170315	UBB	Ubiquitin B	1611
27	132341	RAN	Member RAS oncogene family	1572
31	122674	CCZ1	Vacuolar protein trafficking and biogenesis	1526
33	198668	CALM	Calmodulin	1464
36	134057	CCNB1	Cyclin B1	1384
37	132780	NASP	Nuclear autoantigenic sperm protein	1379
39	173812	EIF1	Eukaryotic translation initiation factor 1	1285
40	221983	UBA52	Ubiquitin A^−52^ residue ribosomal protein fusion product 1	1183
43	076043	REXO2	RNA exonuclease 2	1056
46	115540	MOB4	MOB family member 4, phocein	1007
49	182117	NOP10	NOP10 ribonucleoprotein	962
56	214102	WEE2	WEE1 homolog 2	809
58	162961	DPY30	Histone methyltransferase complex regulatory subunit	790

**Fig. 4:**
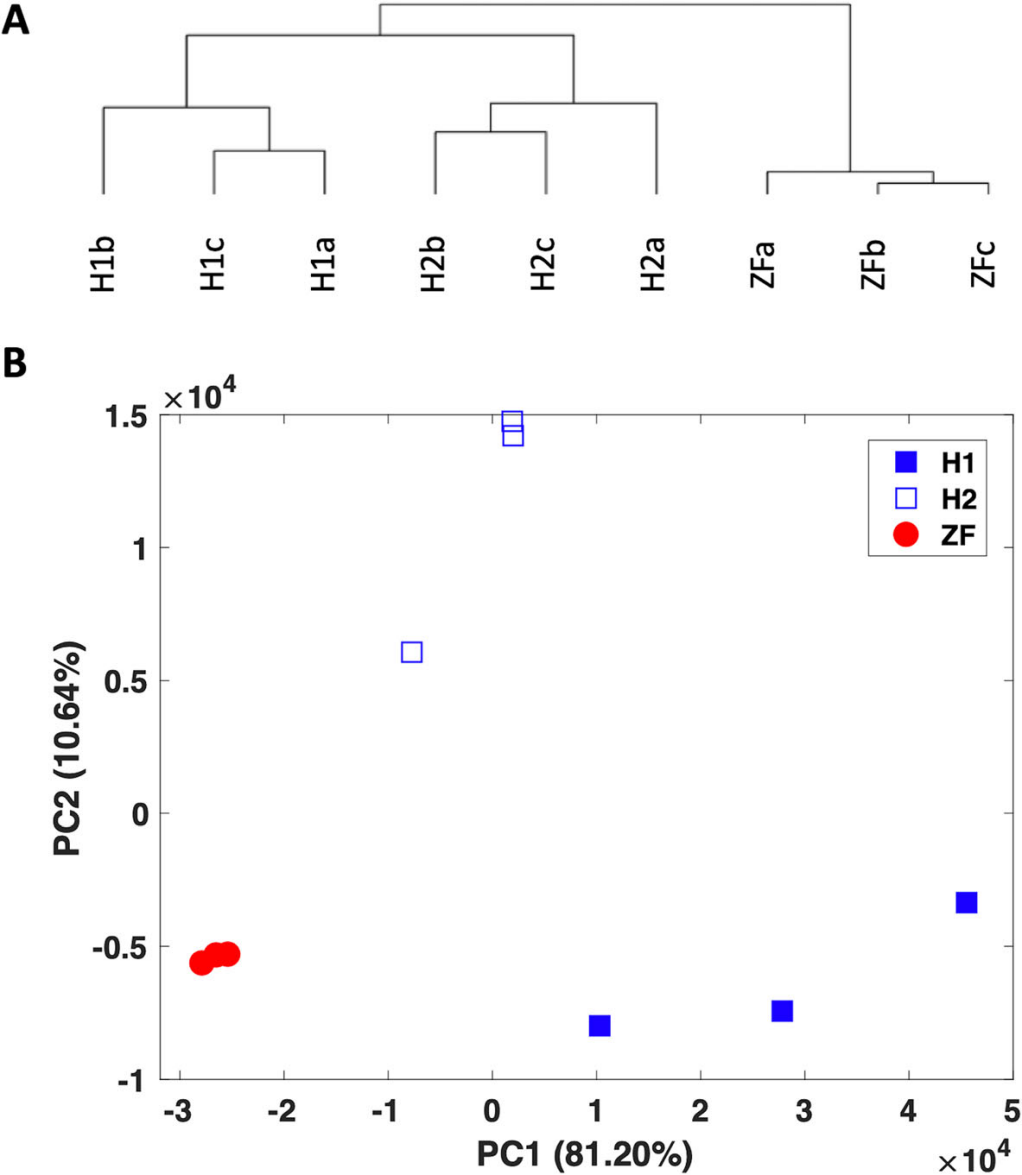
Sample similarity between the oocytes: (**a**) Hierarchical clustering and (**b**) principal components analysis (PCA) of the 9 samples using the 397 highly concordant orthologous genes. In (**b**), the percent variation explained by each PC is shown in parentheses

In order to assess the similarity between the three datasets, we performed hierarchical clustering and principal components analysis (PCA) for the 9 samples using the 397 highly concordant orthologous genes. The results depicted in Fig. 4 show that the two human datasets are more similar to each other than they are to the zebrafish dataset. However, this similarity is not significantly different as the height of the hierarchical clustering branching between the two human datasets is almost as large as the branching between the human and zebrafish datasets. This is also evident in the PCA plot as the three datasets are almost equidistant from each other. Our ANOSIM analysis did not report significant difference between the pairs of datasets (R ~ 0.8, *p* < 0.1) while three-way comparison remained significant (R = 0.93, *p* < 0.005). A similar result was observed in the adonis analysis (pairwise R^2^ ~ 0.71, *p* < 0.1; three-way R^2^ = 0.87, *p* < 0.005). Although from a different organism, the distance between the zebrafish dataset and the two human datasets was not significantly different than the distance between the two human datasets. These results suggest that based on the highly concordant orthologous genes, zebrafish and human oocytes exhibit transcriptomic similarity as the expected organismal differences are not pronounced.

### Functional analysis of the orthologous genes

**Fig. 5:**
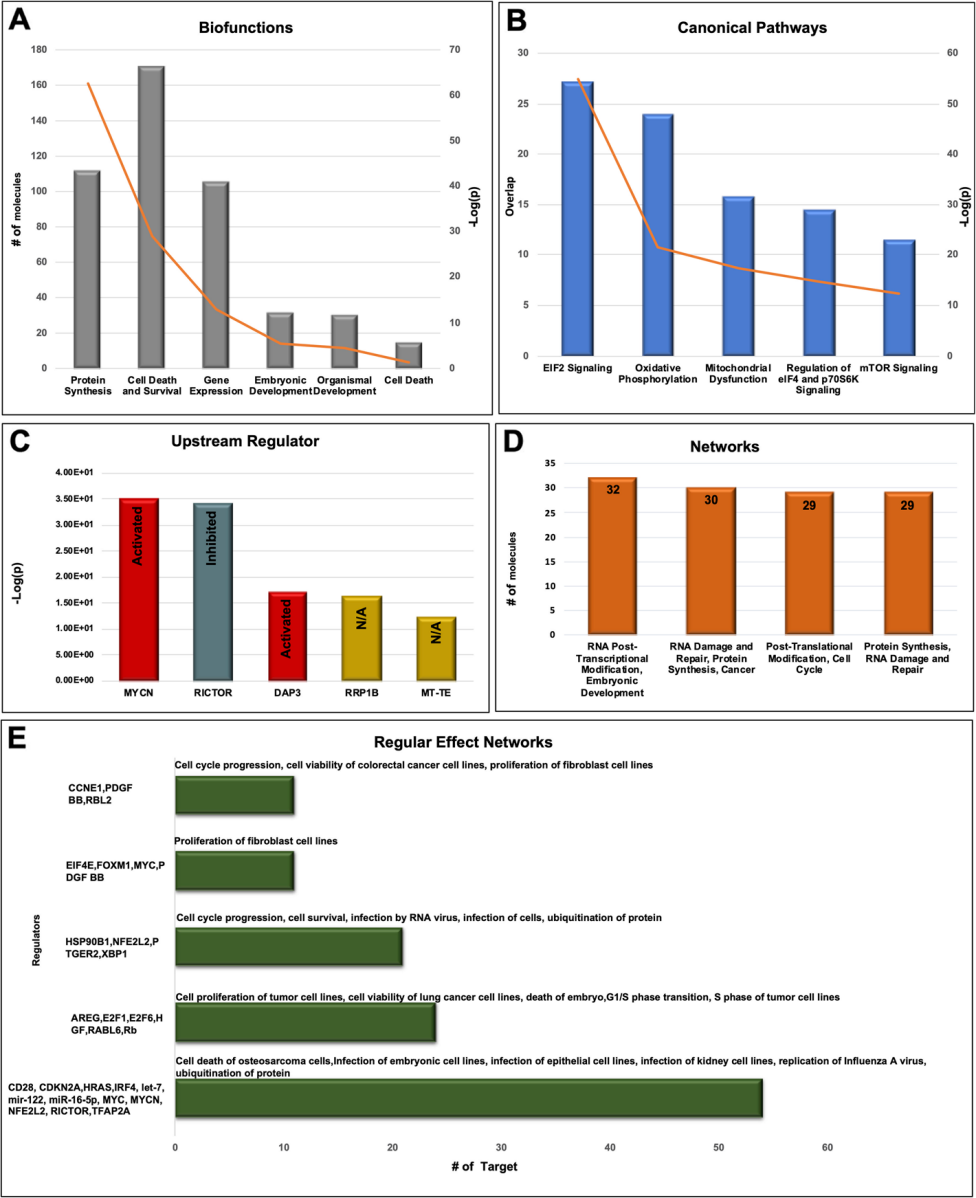
Summary of IPA results based on the 397 highly concordant orthologous genes. **a**, **b** Top Biofunctions and the most significantly enriched Canonical Pathways identified by IPA. Bars represent the number of genes in the functional category or the canonical pathway (primary y-axis) and the orange line represents the significance of the category or the pathway in -Log(*p*-value) (secondary y-axis). **c** Upstream regulators that target a significant portion of the genes in the input list. The inferred activation states of the regulators based on the observed expression of their targets are noted (e.g. an increased expression in targets that are induced by a regulator may imply an “activated” state for the regulator). N/A implies an inconclusive activation state of the regulator. **d** Number of genes and emerging biological functions in the deduced interaction networks that involve input genes. **e** Sets of regulators with a combined target gene set that show concordant enrichment in biological functions. Bars represent the total number of genes targeted by each set of regulators. On each bar, the biological functions that are significantly enriched by the target genes are noted

We used Ingenuity® Pathway Analysis (IPA) (Ingenuity Systems, Redwood City, CA) to analyze the 397 highly concordant orthologous genes and investigated canonical pathways, downstream effects (functions), upstream regulators, regulator effects, and interaction networks. The complete IPA results are cataloged in the supplementary data (Supplementary file 3). In Fig. 5, we present the top members in each category along with associated functions, which is a summary generated by IPA consolidating the detailed categories with the highest significance presented in Supplementary file 3. In the supplementary data, we present the EIF2 signaling pathway, upstream regulator results for MYCN and HNF4A, along with their target molecules, and one gene interaction network highlighting genes involved in embryonic development (Supplementary Figures 1, 2, 3 and 4).

We also analyzed the 397 highly concordant orthologous genes using the EpiFactors database [14] to infer their roles in epigenetic regulation. In Table 2, we list the 36 genes that have been identified in EpiFactors as having an epigenetic function. In the supplementary data (Supplementary file 3), we list the detailed results of the EpiFactors analysis.

**Table 2:** Thirty-six out of the 397 highly concordant orthologous genes with an epigenetic function based on the EpiFactors database. Genes are sorted by decreasing abundance

*Gene ID (ENSG00000+)*	Symbol	Description	Function	SpecificTarget
*130816*	DNMT1	DNA methyltransferase 1	DNA modification	dhC
*132646*	PCNA	Proliferating cell nuclear antigen	Chromatin remodeling	H2A, H2B
*113558*	SKP1	S-phase kinase associated protein 1	Histone modification write cofactor	N/A
*120533*	ENY2	ENY2, transcription and export complex 2 subunit	Histone modification erase cofactor	N/A
*162961*	DPY30	Dpy-30, histone methyltransferase complex regulatory subunit	Histone modification write cofactor	N/A
*187109*	NAP1L1	Nucleosome assembly protein 1 like	Histone chaperone	N/A
*185787*	MORF4L1	Mortality factor 4 like 1	Histone modification read	H4
*132780*	NASP	Nuclear autoantigenic sperm protein	Chromatin remodeling	H1
*276043*	UHRF1	Ubiquitin like with PHD and ring finger domains 1	Histone modification read, Histone modification write cofactor	H3K9me3, H3R2, H3, mCG
*115289*	PCGF1	Polycomb group ring finger 1	Polycomb group (PcG) protein	N/A
*075914*	EXOSC7	Exosome component 7	Scaffold protein, RNA modification	N/A
*075624*	ACTB	Actin beta	Chromatin remodeling cofactor	N/A
*166164*	BRD7	Bromodomain containing 7	Histone modification read	H3K9ac, H3K14ac, H3K8ac
*166913*	YWHAB	Tyrosine 3-monooxygenase/tryptophan 5-monooxygenase activation protein beta	Histone modification erase cofactor	N/A
*149554*	CHEK1	Checkpoint kinase 1	Histone modification write	H3.1
*136938*	ANP32B	Acidic nuclear phosphoprotein 32 family member B	Histone chaperone	H3, H4
*134058*	CDK7	Cyclin dependent kinase 7	Histone modification write	H1
*136518*	ACTL6A	Actin like 6A	Chromatin remodeling cofactor	N/A
*055130*	CUL1	Cullin 1	Chromatin remodeling cofactor	H3K9me3, H3K36me3, H1.4K26me3
*100749*	VRK1	Vaccinia related kinase 1	Histone modification write	H3S10, H3T3
*163875*	MEAF6	MYST/Esa1 associated factor 6	Histone modification write cofactor	H2A, H3K14, H4K5, H4K8, H4K12
*057935*	MTA3	Metastasis associated 1 family member 3	Chromatin remodeling cofactor	N/A
*123737*	EXOSC9	Exosome component 9	Scaffold protein, RNA modification	N/A
*100387*	RBX1	Ring-box 1	Histone modification write cofactor	H3, H4
*151332*	MBIP	MAP 3 K12 binding inhibitory protein 1	Histone modification write cofactor	N/A
*197323*	TRIM33	Tripartite motif containing 33	Histone modification read	H3K9me3, H3K18ac
*119969*	HELLS	Helicase, lymphoid specific	Chromatin remodeling	N/A
*169375*	SIN3A	SIN3 transcription regulator family member A	Histone modification erase cofactor, TF	DNA motif
*133884*	DPF2	Double PHD fingers 2	Chromatin remodeling	N/A
*258315*	C17orf49	Chromosome 17 open reading frame 49	Histone modification read	H3K4me3
*108468*	CBX1	Chromobox 1	Histone modification read	H3K9me3, H3K27me3
*177889*	UBE2N	Ubiquitin conjugating enzyme E2 N	Histone modification write	H2AX
*186591*	UBE2H	Ubiquitin conjugating enzyme E2 H	Histone modification write	H2A, H2B
*139620*	KANSL2	KAT8 regulatory NSL complex subunit 2	Histone modification write cofactor	H5
*100823*	APEX1	Apurinic/apyrimidinic endodeoxyribonuclease 1	DNA modification cofactor	N/A
*167986*	DDB1	Damage specific DNA binding protein 1	Histone modification write	H2A

### Individual oocyte data set characterization

In order to identify functional similarity in the three datasets that is irrespective of orthology, we performed a comparative analysis at the systems level. For this purpose, we identified “highly expressed” genes in each dataset as the genes that have a z-score (based on logged TPM value of “expressed” genes) greater than 1.5 in two out of the three replicates in each study. This resulted in 460 H1, 761 H2, and 901 ZF genes (Supplementary file 4); and the two human datasets had 384 (~ 84%) highly expressed genes in common.

**Fig. 6:**
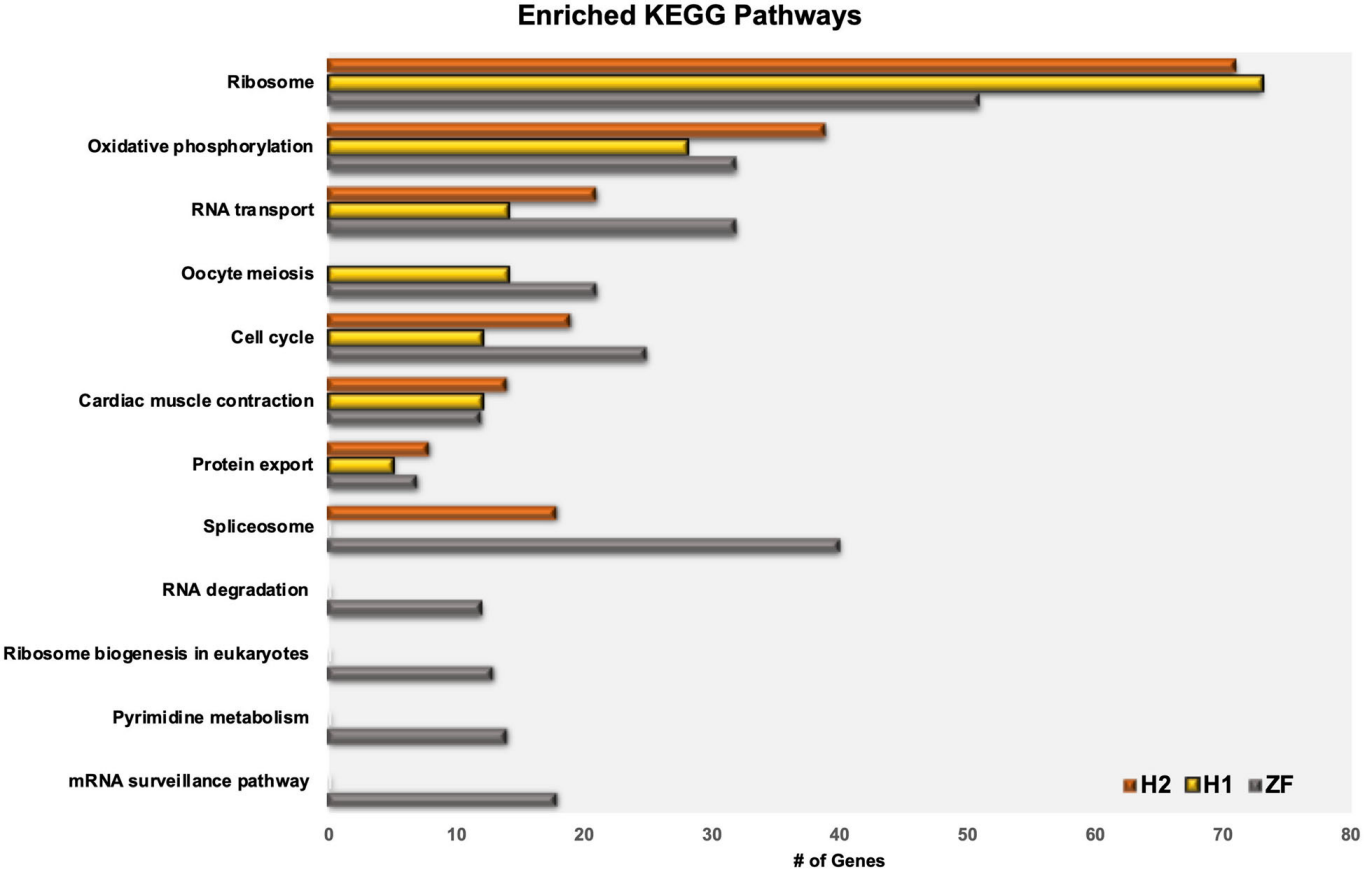
Significantly enriched KEGG pathways based on DAVID functional analysis using genes that are highly expressed in the individual datasets. Bars represent the number of highly expressed genes in the pathway for each data set

We analyzed each of the three highly expressed gene lists separately with the database for annotation, visualization and integrated discovery (DAVID v6.8) [15] to identify enriched Kyoto encyclopedia of genes and genomes (KEGG) pathways [16] and the biological process (BP), molecular function (MF), and cellular component (CC) gene ontology (GO) categories [17]. Detailed results are included in the supplementary data (Supplementary file 4). In Fig. 6, we list the KEGG pathway enrichment analysis results. Our results indicated that the two human datasets showed extreme similarity as expected; moreover, there was significant similarity between the zebrafish and human datasets as well. On average, about 65% of the significantly enriched categories in the zebrafish dataset were also significantly enriched in the human datasets.

### Oocyte-specific gene expression

Although we observe significant concordance in highly expressed genes when orthologous genes are considered, it is possible that functionally important genes, e.g., genes critical in early development, may be expressed at lower levels in the oocyte. We had previously identified human oocyte-specific genes by comparing metaphase II oocytes with a reference consisting of a mixture of total RNA from 10 different normal human tissues not including the ovary [18]. These genes may be expressed at lower quantiles when all of the expressed genes in the oocyte are considered, but they may still have functional significance.

We explored the expression of those human oocyte-specific genes, which mapped to 3493 unique Ensemble Gene IDs, in all three datasets by identifying them on the quantile mapping described in Fig. 3a, b (Supplementary Figure 5, Supplementary file 5). Out of the 3493 human oocyte-specific genes, 2403 (~ 69%) and 3036 (~ 87%) were also expressed in H1 and H2, respectively. Of those 3493 human genes, 2864 (~ 82%) had a high confident ortholog in zebrafish and 2251 (~ 79%) of these 2864 genes were expressed in zebrafish (i.e. had a TPM value greater than 1 in all three replicates).

Our results indicated that the percentage of the orthologous genes, for the functionally important ones, significantly surpassed the percentage of the overall orthologous genes between the two organisms (~ 82% vs. ~ 60%). Furthermore, the functionally important genes that are orthologous are also concurrently highly expressed in the two organisms, which was true for the complete transcriptome as well. For example, the functionally important orthologous genes that are in the top 3 quantiles of expression levels in both organisms (i.e., the top 3 × 3 region of the heatmaps shown in Supplementary Figure 5) contained ~ 20% and ~ 24% of orthologous, functionally important genes expressed in both organisms for H1 and H2 respectively. Both observations were statistically significant (Fisher’s exact test *p*-values < 10^− 14^ and < 10^− 16^ for H1 and H2, respectively). Still, there were genes that were functionally important but were expressed at low levels in the two organisms e.g., orthologous genes that fall beyond the top 3 × 3 region of the heatmaps in Supplementary Figure 5. Nevertheless, although some at low levels, a large subset of the genes that are important for early development (over 80% on average) showed orthology and concordant expression between the two organisms.

## Discussion

A comparative analysis between the zebrafish and the human genome showed high orthology between the two organisms at the gene sequence level [19]. We explored if this similarity was commonly observed at the transcriptome level and investigated the oocyte gene expression across the two organisms. We compared our zebrafish single-cell RNA-seq data to two published single-cell RNA-seq datasets from human oocytes. All three datasets have used three single cell metaphase II (MII) oocytes at the same stage, which are comparable across organisms, considered fully matured and ready to be fertilized. All three datasets have been obtained using standard RNA-seq protocols and our analysis began with the raw data obtained in the three experiments using the same computational workflow to prevent any variations. We found that majority of the genes expressed in the human oocyte have an ortholog that is also expressed in the zebrafish oocyte. However, when we divided the expressed genes in each data set into ten quantiles based on their level of expression, we found that the degree of concordance of both species increased significantly for highly expressed genes. Our results indicate that the number of high-confidence orthologous genes expressed in both organisms was about four times the number of high-confidence orthologous genes expressed in only one organism.

Among the genes expressed in the zebrafish egg, and not expressed in the human oocytes or with no orthologue in human, we found members of the family of the elongation of very long-chain fatty acid (Elovl) genes ELOVL1A, 8B, 7B, 6 L, 1B, and 4B; and those highly expressed were ORG, CLDND, CLDNG, and HMGB3B, in agreement with previous reports [20–23]. Also, interestingly, several pluripotency-associated genes appear specifically in the zebrafish egg, as NANOG, Pou5f3, GDF3, KLF2 and KLF5, opening a venue to use the zebrafish oocyte as a model for studying the role of maternal “pluripotency” genes that can have a significant role, but different than those described so far in mammals, during post-fertilization reprogramming [24].

We performed IPA analysis on the highly concordant orthologous genes and found that EIF2 signaling and oxidative phosphorylation were the top two activated “canonical pathways” based on statistical significance assessing their enrichment (*p* < 10^− 20^). For “upstream regulators,” the top ranked were the protooncogene MYCN (*p* < 10^− 35^), as activated, and RICTOR (*p* < 10^− 34^), as inhibited where the regulators are ranked by the significance of overlap between their targets and the input gene list to the IPA. Genes associated with regulation of cell cycle progression were involved in regulator effect networks analysis, which identify a congruent theme for the targets of upstream regulators that are among the input genes (the highly concordant orthologous genes). Furthermore, the “top active networks” identified by IPA (based on statistical significance of the involvement of the input genes in the networks using Fisher’s exact test) were RNA post-transcriptional modification and embryonic development (*p* < 10^− 50^). Based on our enrichment analysis using DAVID, “spliceosome,” “oxidative phosphorylation,” “RNA transport,” and “ribosome” were among the KEGG pathways common to all three datasets further underlining the functional similarity between the oocytes of the two organisms. Details of the IPA and DAVID functional analysis can be found in Supplementary files 3 and 4.

We used the EpiFactors database to identify genes that are known to be involved in epigenetic modifications; and we found a high number of the highly concordant orthologous genes to be listed (~ 10%). The list includes well characterized genes, such as DNMT1, PCNA, and UHRF1. Also listed was DPY30, which has recently been characterized in the context of embryonic development and required for pluripotency maintenance [25]. Other epigenetic chromatin reprogramming factors, such as TET3 digoxygenase or histone modifier factors (HDAC1, HDAC8, SIRT1, SIRT7, HAT1, or TRAM1), with a crucial role in the zygote following fertilization and nuclear reprogramming also appear listed, further confirming functional concordance between species [26, 27]. Similarly, the expression of MTA3 in all datasets is thought provoking. MTA3 belongs to the family of metastasis associated proteins, known for their role in cancer progression; however, there are no studies describing its role in gametes or embryonic development [28].

When we explored the distribution of oocyte-specific genes in order to assess the levels of expression and orthology for genes that may be important in early development, we observed an improved agreement between the two organisms. Both the percentage and expression levels of the orthologous genes for the oocyte-specific genes were higher than those observed when all of the genes were considered. Therefore, the amount and degree of agreement between the two organisms increased for functionally crucial genes, further validating the use of zebrafish as a model organism in developmental biology.

## Conclusions

In order to understand the differences and similarities between human and zebrafish matured oocytes, we performed RNA-seq of individual zebrafish eggs and compared them to two different datasets of individual human oocytes at the same developmental stage. Our results indicate that the degree of orthology between the expressed genes in the two organisms is significantly larger for highly expressed genes. Moreover, the functional relevance based on gene expression in the two organisms’ oocytes shows high concordance. Our study compares the observed transcriptome of the metaphase II oocytes, including genes that are deposited in the oocytes for late usage during the development. Our results represent the similarity between the two organisms for the given stage and indicates not only high orthology but high functional similarity in their transcriptomes.

Despite the significant evolutionary distance between human and zebrafish, the mature female gametes of both species show significant similarities in gene expression. The results of these functional analyses underscore the use of zebrafish as a valuable animal model and provide evidence for future hypothesis-driven experiments related to germ cell development, gametogenesis, and epigenetic inheritance, among others. Future studies may involve comparative transcriptome analysis on other cell types.

## Methods

### Zebrafish maintenance

All experimental protocols were approved by the Michigan State University Institutional Animal Care and Use Committee, the Spanish Institutional Animal Use and Care Committee regulations and the Regional Andalusian Government (code A/ES/14/43). All animal methods were carried out in accordance with standard practices described in ‘The Zebrafish Book’ and by ZM Varga 2016 [29, 30]. The zebrafish used in this study were obtained from the Zebrafish International Resource Center (ZIRC; http://zebrafish.org). After the study, animals were euthanized according with our American Association for Accreditation of Laboratory Animal Care (AAALAC) approved protocol: Fish were euthanized in sodium bicarbonate buffered tricaine methanesulphonate (MS-222) at a concentration of 250 mg/L according to American Veterinary Medical Association (AVMA) guidelines on euthanasia. This is administered by adding directly to the water to which fish are acclimated. Solution was made fresh for every 10 fish to ensure that the dosage and buffering remain effective. Death was confirmed by absence of heartbeat and corneal reflex.

### Zebrafish egg isolation

The F1 zebrafish, a cross between the Tübinguen and AB lines of zebrafish were used as egg donors. Only females proven to give rise to > 90% of fertilized eggs at 24 h post fertilization (hpf) were chosen and subsequently used for egg collection. The eggs were obtained from the same female to minimize variability. Equipment and reagents were prepared beforehand as previously described [31]. Females were transferred into a beaker with a sedation solution (0.02% w/v tricaine solution or MS222). When movement ceased, we rinsed the fish in clean water. Each female was set on a folded Kimwipe® paper in an upturned position so that the genital opening was easily accessible. The abdominal area surrounding the genitalia was dried with Kimwipe paper. A small amount of pressure was gently applied to two sides of the belly using a rounded-edge glass rod and a fingertip. Inactivated eggs were collected in Chinook salmon ovarian fluid (CSOF) to maintain the metaphase-arrested stage of the eggs [32]. The morphological appearance of each egg was examined, and only high-quality eggs were used for sequencing, as previously described [31–33]. Eggs were rinsed twice in 0.5% bovine serum albumin in Hank’s Balanced Salt Solution to wash off the CSOF. Then, the eggs were swiftly rinsed in RNAse-free phosphate buffered saline (PBS) three times and immediately transferred to a 1.5-ml tube –one oocyte per tube– with the least amount of PBS left, snap frozen using liquid nitrogen for 1 min of immersion, and then stored at − 80 °C until RNA isolation.

### RNA-seq experimental procedure

RNA isolation and purification were performed using Direct-zolTM RNA MicroPrep according with manufacturer instructions (Zymo Research, Irvine, CA) on three individual oocytes. Briefly, frozen eggs were thawed at room temperature and resuspended in Tri Reagent, followed by addition of 95% ethanol, DNase I treatment, and centrifugation in an IC column [34]. Final RNA elution was performed using DNase/RNase-free water. Total RNA was processed for library construction by Cofactor Genomics (St. Louis, MO) according to the following procedure: Briefly, total RNA was reverse-transcribed using an Oligo (dT) primer, and limited cDNA amplification was performed using the SMARTer® Ultra® Low Input RNA Kit for Sequencing – v4 (Takara Bio USA, Inc., Mountain View, CA). The resulting full-length cDNA was fragmented and tagged, followed by limited PCR enrichment to generate the final cDNA sequencing library (Nextera® XT DNA Library Prep, Illumina, San Diego, CA). Libraries were sequenced as single-end 75 base pair reads on an Illumina NextSeq500 following the manufacturer’s instructions.

### Human datasets

We used two single-cell RNA-seq datasets from human oocytes (labeled H1 and H2). For dataset H1 (Gene Expression Omnibus (GEO) accession number GSE44183), oocytes were obtained from female patients that were between 26 and 35 years old. Oocytes were vitrified and thawed at the time of RNA isolation. Only oocytes that had an intact cell membrane and zona pellucida were used (*n* = 3) [12]. For dataset H2 (GEO accession number GSE110798), oocytes were also obtained from women undergoing fertility treatments. RNA isolation was performed in in vivo matured oocytes immediately after follicle aspiration (*n* = 3) [13].

### RNA-seq analysis

We generated single-end, single-cell RNA-seq data for three zebrafish oocytes (labeled ZF). The average read length was 75 base pairs (bp), and the average number of reads per sample was ~ 46.3 million. Both human datasets were processed using paired-end sequencing. The first dataset, H1, had an average read length of 90 bp and an average number of reads per sample of ~ 37.4 million (~ 18.7 million fragments) [12]. The average read length for H2 samples was 100 bp with an average number of reads per sample of ~ 59.9 million (~ 30.0 million fragments) [13].

We processed all three datasets using FastQC (v. 0.11.5) and Trimmomatic (v 0.38) [35, 36]. Overrepresented sequences due to experimental artifacts (e.g., adapters and similar technical sequences) were identified and subsequently removed. Trimmomatic was used in the palindrome mode, based on default alignment detection and scoring parameters. Low quality bases were trimmed using the maximum information quality filtering followed by a minimum average read quality threshold of 25. Following technical sequence and low-quality base removal, reads that were shorter than 40 bp were filtered out. Sequencing quality metrics for all three datasets (both for raw and trimmed+filtered reads) were assessed using FQStat, which resulted in passing values with respect to the default quality control thresholds identified therein [37].

Transcript quantification was done using Salmon (v. 0.8.2) with default parameters based on Ensembl database annotations [38, 39]. Salmon uses sample-specific models, such as correction for guanine-cytosine (GC) content bias, that improve the accuracy of transcription abundance estimates. We used transcripts per million (TPM) in Salmon’s output as the normalized relative abundance measure employed in our downstream analysis. Gene-level abundance estimates were obtained using the R package (tximport) as gene-level signal summaries have shown to be the more robust statistic [40].

### Gene expression analysis

For each dataset, we identified genes that are “expressed” in a dataset as the genes that had a TPM value greater than 1 in all three replicates used in the dataset. In order to identify genes that are “highly expressed” in a dataset, we calculated the z-score of the genes (in a dataset) based on the logged TPM values of the “expressed” genes. If the gene’s z-score in a dataset exceeded 1.5 in at least two out of the three replicates, we identified the gene as “highly expressed” in this dataset. Orthologous genes between the two organisms were obtained from the Ensembl database along with the confidence assessment in orthology, which is a binary classification: 1 (high) or 0 (low).

Genes expressed in a human dataset were contrasted with the genes expressed in the zebrafish dataset using quantile mapping. Genes expressed in a human data set were divided into ten quantiles, and genes in each quantile were mapped through orthology to the ten expression quantiles identified for the genes expressed in the zebrafish dataset. For each human quantile, we further assessed the genes that did not have an orthologue in zebrafish and the genes that had an orthologue in zebrafish but were not identified as expressed in the zebrafish dataset. Similarly, for each zebrafish quantile, we identified genes that did not have an orthologue in human and genes that had an orthologue in human but were not identified as expressed in the human dataset. Quantile mapping was done for the two human datasets as well, in order to assess their similarity in gene expression.

Clustering of samples was done using the unweighted pair group method with arithmetic-mean (UPGMA) method with Pearson’s correlation as the distance measure [41]. The expression data matrix was row normalized prior to the application of average linkage clustering. Samples were also used for principal components analysis (PCA), which is a dimension reduction technique that represents samples along orthogonal PCs (axis) that decreasingly capture the variance in the underlying data [42]. Plotting the samples along the PCs reflect the closeness between the samples and inherently imply an alternative clustering view for the samples. Both the hierarchical clustering and PC analyses were done using MATLAB® (The MathWorks, Inc., Natick, MA). Analysis of similarity (ANOSIM) [43] and adonis (permutational multivariate analysis of variance using distance matrices) [44] tests were applied to PCA results using the vegan package (v. 2.3–5 and v. 2.4–2) in R (v. 3.5.3) to evaluate the differences between the three datasets.

### Systems biology analysis

Gene sets of interest were analyzed using the database for annotation, visualization and integrated discovery (DAVID v6.8), the Ingenuity® pathway analysis (IPA) (Ingenuity Systems, Redwood City, CA), and the EpiFactors database [14, 15]. Functional analysis of the gene lists was done using DAVID based on the biological process (BP), molecular function (MF), and cellular component (CC) gene ontology (GO) categories and the Kyoto encyclopedia of genes and genomes (KEGG) pathways [15, 16]. The expression analysis systematic explorer (EASE) score was used to assess over represented categories that are biologically relevant and warrant further investigation. The EASE score is the upper bound of the distribution of jackknife iterative resampling of Fisher exact probabilities with Bonferroni multiple testing correction. Categories containing low numbers of genes are under weighted so that the EASE score is more robust than the Fisher exact test. The EASE score is a significance level with smaller EASE scores indicating increasing confidence in over representation. We picked GO categories and KEGG pathways that have EASE scores of 0.05 or lower as significantly overrepresented.

We employed IPA, which uses Ingenuity® Pathways Knowledge Base (IPKB), a manually curated knowledgebase based on scientific literature that involves biological interactions and functional annotations for genes and gene products. Given a gene list, IPA uses enrichment analysis-based approaches to identify canonical pathways, downstream effects, upstream regulators, regulator effects, and interaction networks that best explain the observed expression levels [45, 46]. In the canonical pathways and downstream effects analysis, known pathways or functions that involve a significant number of the input genes are identified. The upstream regulators approach identifies transcription factors, microRNAs, kinases, compounds, drugs, etc., that are known to regulate a significant portion of the input genes. The regulator effects analysis combines the upstream regulator and downstream effects analyses to develop a causal hypothesis combining upstream regulators with supposedly regulated input genes that are significantly represented in a biological function. Finally, the interaction network analysis identifies input (and other) genes that are highly connected based on literature and overlays a number of features, such as expression, function, drug, etc., regarding the genes in the network. IPA also calculates the “state” of the individual analysis target (e.g., “activating” or “inhibiting”) based on the observed expression changes and the known causal relationships from the literature.

In order to investigate the epigenetic involvement of the gene lists of interest, we cross-referenced them with the EpiFactors database. EpiFactors is a manually curated database that catalogues genes, proteins, and complexes that are involved in epigenetic regulation. Functional and relational annotation is provided for regulators and their targets, which may be used to associate gene expression patterns with epigenetic regulation.

## Supplementary information

**Additional file 1: Figure S1.** EIF2 Signaling. Eukaryotic initiation factor 2 (eIF2) canonical pathway that is significantly enriched by the 397 highly concordant and high-confident orthologous genes between human and zebrafish oocytes. Members of the 397-gene set are indicated in red. Numbers below highlighted genes show log10 of the average transcripts per million (TPM) values. If a node is a complex/group, then the TPM value is omitted. Intensity of the red color is proportional to the degree of up-regulation (i.e. high TPM). **Figure S2.** HNF4a targets. Targets of the upstream regulator hepatocyte nuclear factor 4 alpha (HNF4A) that are among the 397 highly concordant and high-confident orthologous genes between human and zebrafish oocytes. Numbers below the target genes show log10 of the average transcripts per million (TPM) values. Intensity of the red color is proportional to the degree of up-regulation (i.e. high TPM). **Figure S3.** MYCN targets. Targets of the upstream regulator MYCN that are among the 397 highly concordant and high-confident orthologous genes between human and zebrafish oocytes. Numbers below the target genes show log10 of the average transcripts per million (TPM) values. Intensity of the red color is proportional to the degree of up-regulation (i.e. high TPM). **Figure S4.** Gene interaction network. Interaction network among a subset of the 397 highly concordant and high-confident orthologous genes between human and zebrafish oocytes. Numbers below the target genes show log10 of the average transcripts per million (TPM) values. Intensity of the red color is proportional to the degree of up-regulation (i.e. high TPM). Genes that belong to the “Embryonic Development” functional category are highlighted with a pink outline. **Figure S5.** Identification of the 3,493 oocyte-specific genes on the quantile mapping described in Fig. 3A, B. (A) H1 vs. ZF, (B) H2 vs. ZF. Column 11: oocyte-specific genes that are expressed in human, have a high-confident orthologue in zebrafish, but are not expressed in zebrafish; Column 12: oocyte-specific genes that are expressed in human but do not have a high-confidence orthologue in zebrafish. Row 11: oocyte-specific genes that are expressed in zebrafish, have a high-confident orthologue in human, but are not expressed in human.

**Additional file 2: Supplementary file 1.** Transcripts per million (TPM) values and distribution for all nine samples in the three data sets (H1, H2, ZF) with respect to gene types.

**Additional file 3: Supplementary file 2.** List of genes described by the quantile mapping data matrices (Fig. 3). For each location (given row/column position in the quantile mapping matrix), genes are listed along with their description and TPM values in the samples involved in the mapping.

**Additional file 4: Supplementary file 3.** List of 397 highly concordant and high-confident orthologous genes between human and zebrafish oocytes and the functional and systems biology analysis results for theses 397 genes. Ingenuity Pathway Analysis (IPA) results show the list of significant canonical pathways, biological/toxicological functions, upstream regulators, regulator effects and gene interaction networks. EpiFactors database analysis identifies the genes among the 397 that have been shown to be involved in epigenetic regulation along with their targets.

**Additional file 5: Supplementary file 4.** Lists of highly expressed genes in the three data sets (H1, H2, ZF) and statistically significantly overrepresented Kyoto Encyclopedia of Genes and Genomes (KEGG) pathways, and Gene Ontology (GO) Biological Process (BP), Molecular Function (MF), and Cellular Component (CC) categories based on Database for Annotation, Visualization and Integrated Discovery (DAVID v6.8) analysis. DAVID analysis is done separately for the three highly expressed gene lists and a comparative analysis of these individual results are presented.

**Additional file 6: Supplementary file 5.** Oocyte-specific genes that appear in the list of genes described by the quantile mapping data matrices (Fig. 3), which were summarizied in Supplementary file 2.

## Data Availability

The dataset supporting the conclusions of this article is available in NCBI’s SRA database under the accession ID: PRJNA524247 (https://www.ncbi.nlm.nih.gov/Traces/study/?acc=PRJNA524247).

## Abbreviations

BP: Biological process
CC: Cellular component
CLDND: Claudin d
CLDNG: Claudin g
DAVID: Database for annotation, visualization and integrated discovery
DNMT1: DNA methyltransferase 1
DPY30: Dpy-30 histone methyltransferase complex regulatory subunit
EIF2: Eukaryotic initiation factor 2
ELOVL: Elongation of very long-chain fatty acid
GDF3: Growth differentiation factor 3
GO: Gene ontology
HAT1: Histone acetyltransferase 1
HDAC1: Histone deacetylase 1
HDAC8: Histone deacetylase 8
HMGB3B: High mobility group box 3b
HNF4A: Hepatocyte nuclear factor 4 alpha
IPA: Ingenuity® pathway analysis
KEGG: Kyoto encyclopedia of genes and genomes
KLF2: Kruppel like factor 2
KLF5: Kruppel like factor
lincRNA: Long intervening noncoding RNA
MF: Molecular function
miRNA: Micro RNA
misc_RNA: Miscellaneous other RNA
MTA3: Metastasis associated 1 family, member 3
Mt-rRNA: Mitochondrial ribosomal RNA
ORG: Oogenesis-related gene
PCA: Principal components analysis
PCNA: Proliferating cell nuclear antigen
Pou5f3: POU domain, class 5, transcription factor 3
RICTOR: Regulatory-associated protein of mechanistic target of rapamycin (mTOR) (RPTOR) independent companion of mTOR complex 2
rRNA: Ribosomal RNA
scRNA: Small cytoplasmic RNA
siRNA: Small interfering RNA
SIRT1: Sirtuin 1
SIRT7: Sirtuin 7
snoRNA: Small nucleolar RNA
snRNA: Small nuclear RNA
TET3: Tet methylcytosine dioxygenase 3
TPM: Transcripts per million
TRAM1: Translocation associated membrane protein 1
UHRF1: Ubiquitin like with PHD and ring finger domains 1
