# Supplementary material for: Comparative analysis of single-cell transcriptomics in human and Zebrafish oocytes
Source: BMC Genomics. 2020 Jul 8;21:471. doi: 10.1186/s12864-020-06860-z (PMC7346435; doi:10.1186/s12864-020-06860-z)
Supplement: Supplementary file 1 — Additional file 1: Figure S1. EIF2 Signaling. Eukaryotic initiation factor 2 (eIF2) canonical pathway that is significantly enriched by the 397 highly concordant and high-confident orthologous genes between human and zebrafish oocytes. Members of the 397-gene set are indicated in red. Numbers below highlighted genes show log10 of the average transcripts per million (TPM) values. If a node is a complex/group, then the TPM value is omitted. Intensity of the red color is proportional to the degree of up-regulation (i.e. high TPM). Figure S2. HNF4a targets. Targets of the upstream regulator hepatocyte nuclear factor 4 alpha (HNF4A) that are among the 397 highly concordant and high-confident orthologous genes between human and zebrafish oocytes. Numbers below the target genes show log10 of the average transcripts per million (TPM) values. Intensity of the red color is proportional to the degree of up-regulation (i.e. high TPM). Figure S3. MYCN targets. Targets of the upstream regulator MYCN that are among the 397 highly concordant and high-confident orthologous genes between human and zebrafish oocytes. Numbers below the target genes show log10 of the average transcripts per million (TPM) values. Intensity of the red color is proportional to the degree of up-regulation (i.e. high TPM). Figure S4. Gene interaction network. Interaction network among a subset of the 397 highly concordant and high-confident orthologous genes between human and zebrafish oocytes. Numbers below the target genes show log10 of the average transcripts per million (TPM) values. Intensity of the red color is proportional to the degree of up-regulation (i.e. high TPM). Genes that belong to the “Embryonic Development” functional category are highlighted with a pink outline. Figure S5. Identification of the 3,493 oocyte-specific genes on the quantile mapping described in Fig. 3A, B. (A) H1 vs. ZF, (B) H2 vs. ZF. Column 11: oocyte-specific genes that are expressed in human, have a high-confident ortholo [file 12864_2020_6860_MOESM1_ESM.docx]

**Supplementary Information for**

**Comparative Analysis of Single-Cell Transcriptomics in Human and Zebrafish Oocytes**

Handan Can,^1^ Sree K. Chanumolu,^1^ Elena Gonzalez-Muñoz,^2^ Sukumal Prukudom,^3^ Hasan H. Otu,(*)^1^ Jose B. Cibelli(*)^4^

1. Department of Electrical and Computer Engineering, University of Nebraska-Lincoln, Lincoln, NE 68588

2. LARCEL, Andalusian Laboratory of Cell Reprogramming (LARCel), Andalusian Center for Nanomedicine and Biotechnology-BIONAND, 29590 Málaga; and Department of Cell Biology, Genetics and Physiology, University of Málaga and; Networking Research Center on Bioengineering, Biomaterials and Nanomedicine, (CIBER-BBN), 29071 Málaga, Spain.

3. Center for Advanced Studies for Agriculture and Food, Kasetsart University Institute for Advanced Studies, Kasetsart University, Bangkok 10900, Thailand (CASAF, NRU-KU).

4. Departments of Animal Science and Large Animal Clinical Sciences, Michigan State University, East Lansing MI 48824, USA.

(*) co-corresponging authors, [hotu2@unl.edu](mailto:hotu2@unl.edu) and [cibelli@msu.edu](mailto:cibelli@msu.edu)

**Description of Supplementary Excel Files**

Supplementary File 1: Transcripts per million (TPM) values and distribution for all nine samples in the three data sets (H1, H2, ZF) with respect to gene types.

Supplementary File 2: List of genes described by the quantile mapping data matrices (Figure 2). For each location (given row/column position in the quantile mapping matrix), genes are listed along with their description and TPM values in the samples involved in the mapping.

Supplementary File 3: List of 397 highly concordant and high-confident orthologous genes between human and zebrafish oocytes and the functional and systems biology analysis results for theses 397 genes. Ingenuity Pathway Analysis (IPA) results show the list of significant canonical pathways, biological/toxicological functions, upstream regulators, regulator effects and gene interaction networks. EpiFactors database analysis identifies the genes among the 397 that have been shown to be involved in epigenetic regulation along with their targets.

Supplementary File 4: Lists of highly expressed genes in the three data sets (H1, H2, ZF) and statistically significantly overrepresented Kyoto Encyclopedia of Genes and Genomes (KEGG) pathways, and Gene Ontology (GO) Biological Process (BP), Molecular Function (MF), and Cellular Component (CC) categories based on Database for Annotation, Visualization and Integrated Discovery (DAVID v6.8) analysis. DAVID analysis is done separately for the three highly expressed gene lists and a comparative analysis of these individual results are presented.

Supplementary File 5: Oocyte-specific genes that appear in the list of genes described by the quantile mapping data matrices (Figure 2), which were summarizied in Supplementary File 6.

**The following legends are used in the IPA software that describe the types of shapes, links, and colors used in the supplementary figures.**


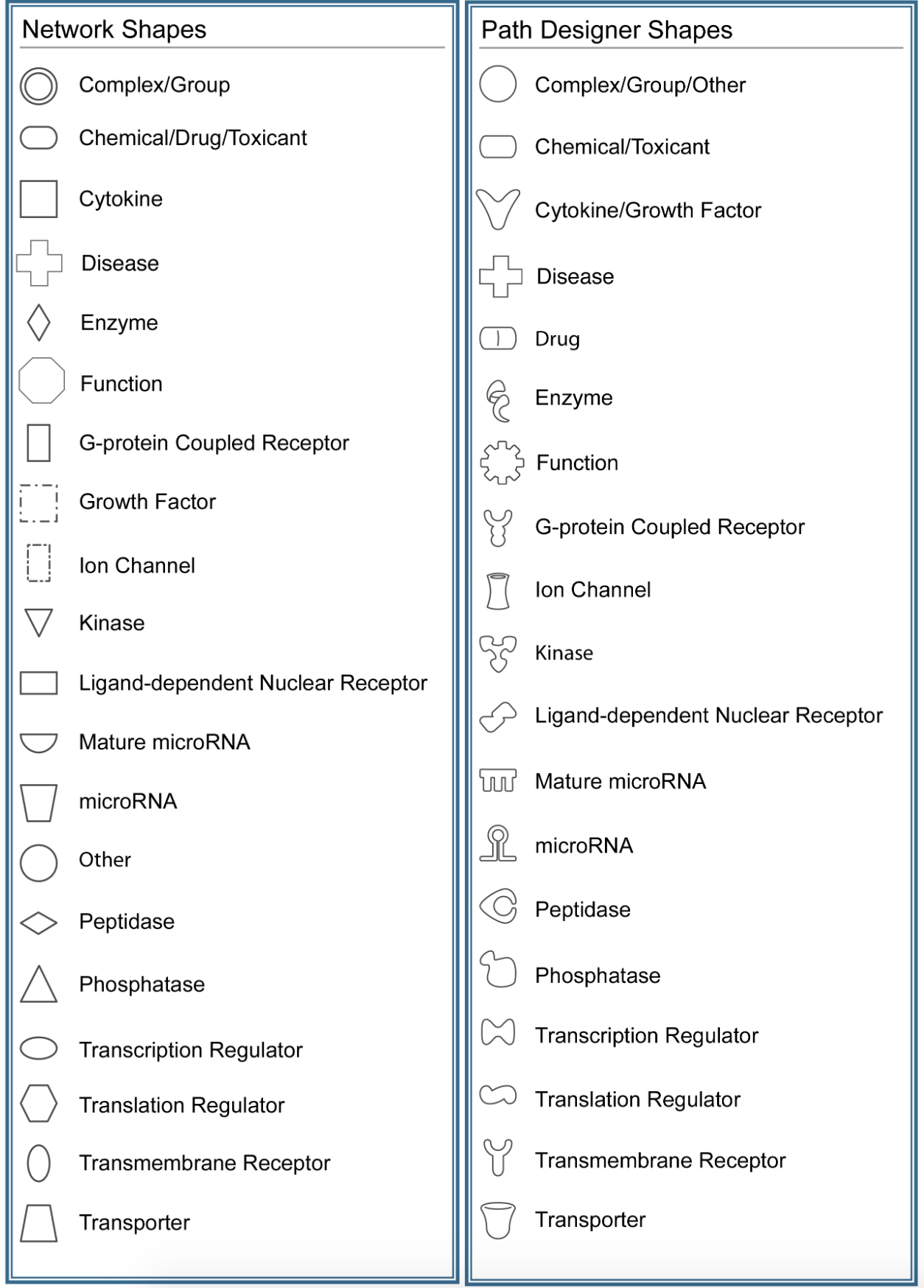


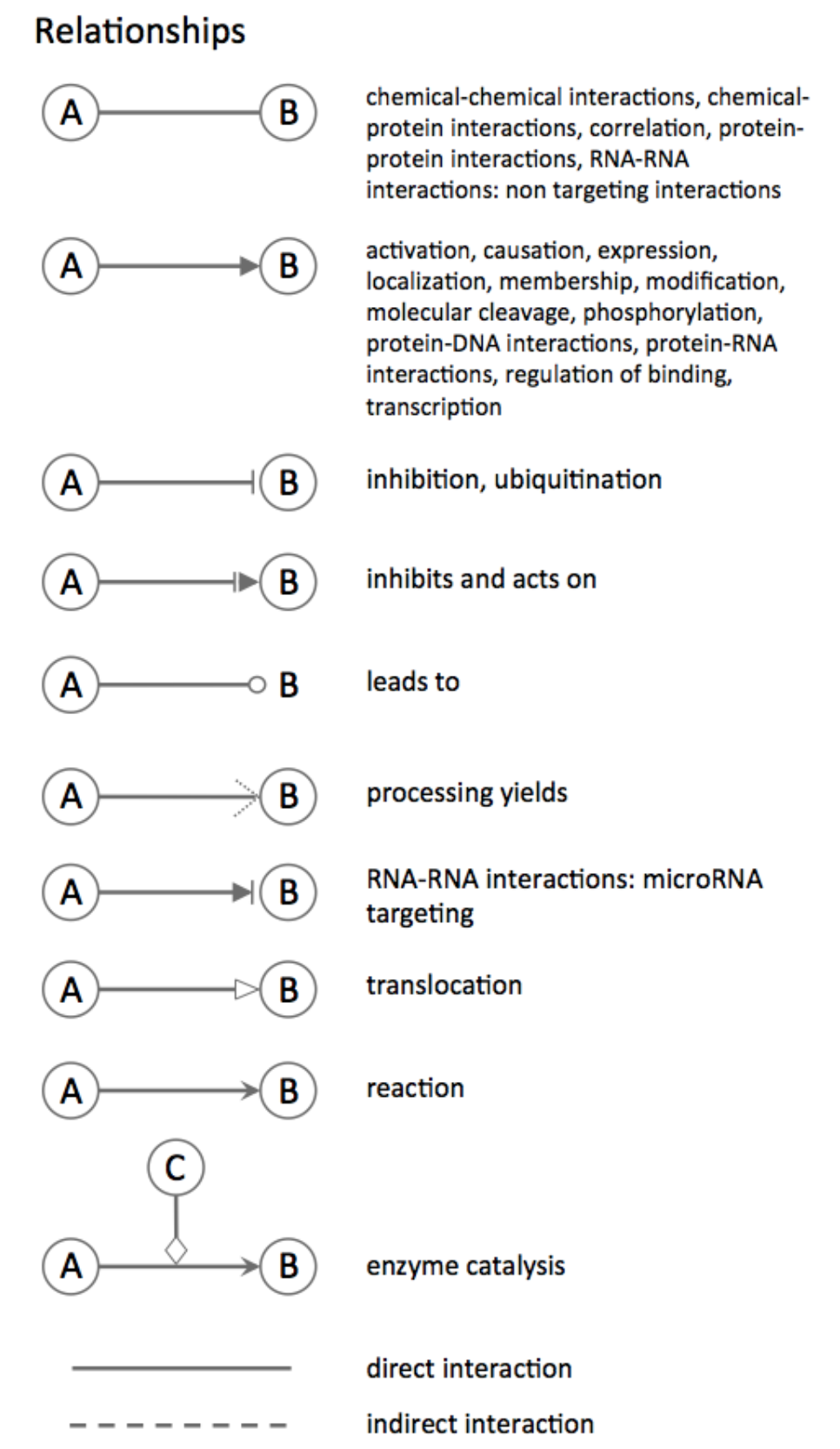


**Supplementary Figures**

**
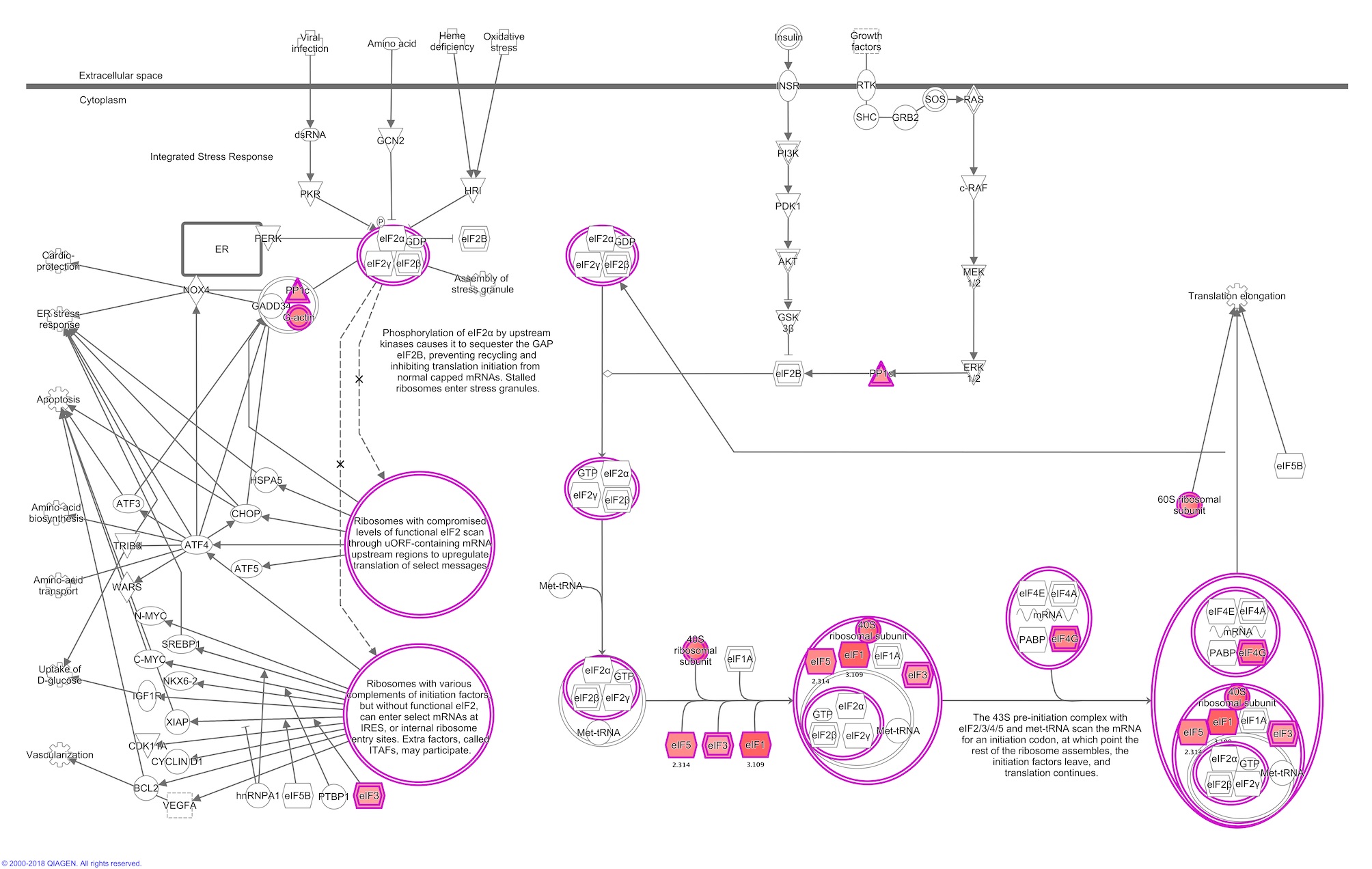
**

Supplementary Figure 1: EIF2 Signaling. Eukaryotic initiation factor 2 (eIF2) canonical pathway that is significantly enriched by the 397 highly concordant and high-confident orthologous genes between human and zebrafish oocytes. Members of the 397-gene set are indicated in red. Numbers below highlighted genes show log10 of the average transcripts per million (TPM) values. If a node is a complex/group, then the TPM value is omitted. Intensity of the red color is proportional to the degree of up-regulation (i.e. high TPM).


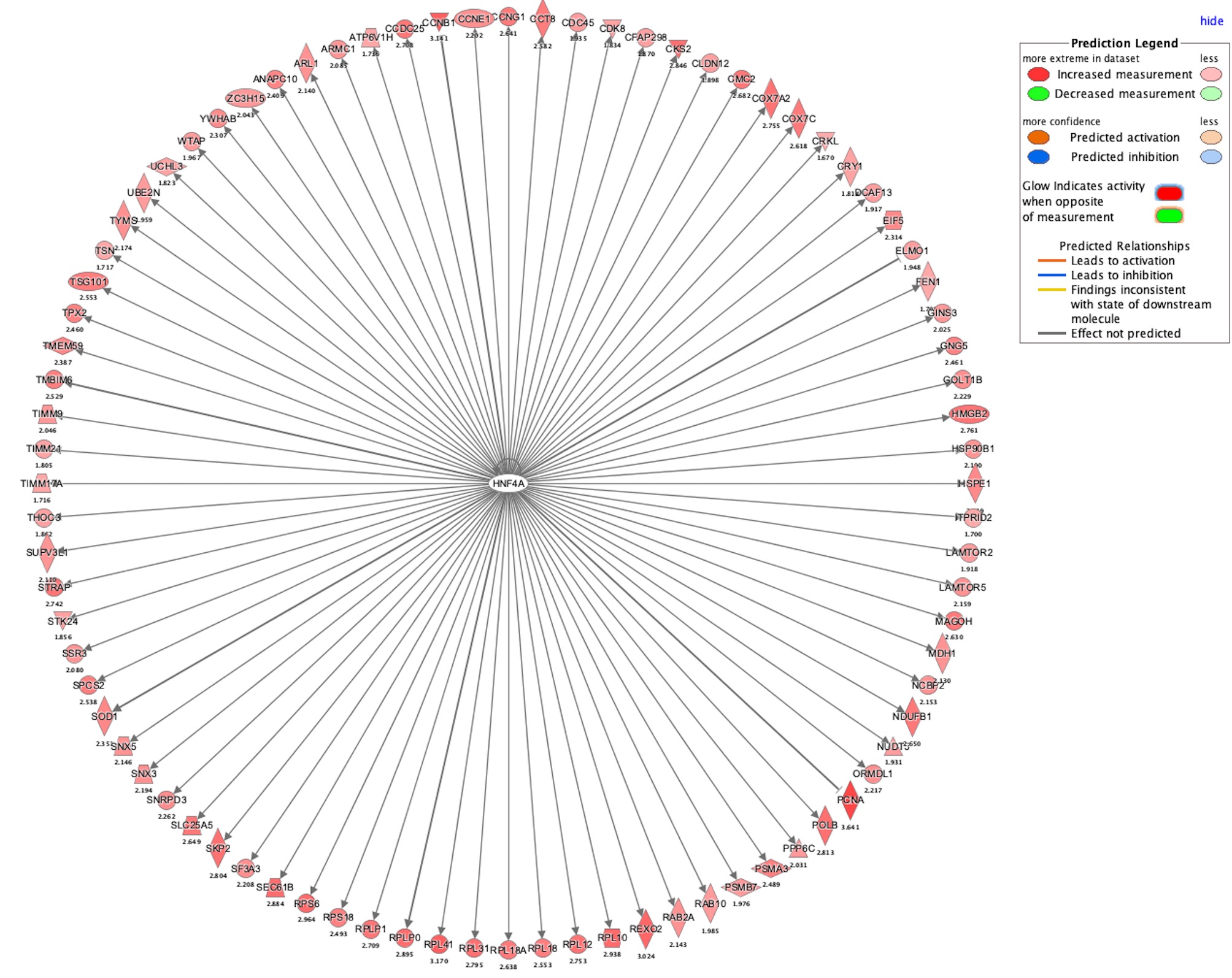


Supplementary Figure 2: HNF4a targets. Targets of the upstream regulator hepatocyte nuclear factor 4 alpha (HNF4A) that are among the 397 highly concordant and high-confident orthologous genes between human and zebrafish oocytes. Numbers below the target genes show log10 of the average transcripts per million (TPM) values. Intensity of the red color is proportional to the degree of up-regulation (i.e. high TPM).


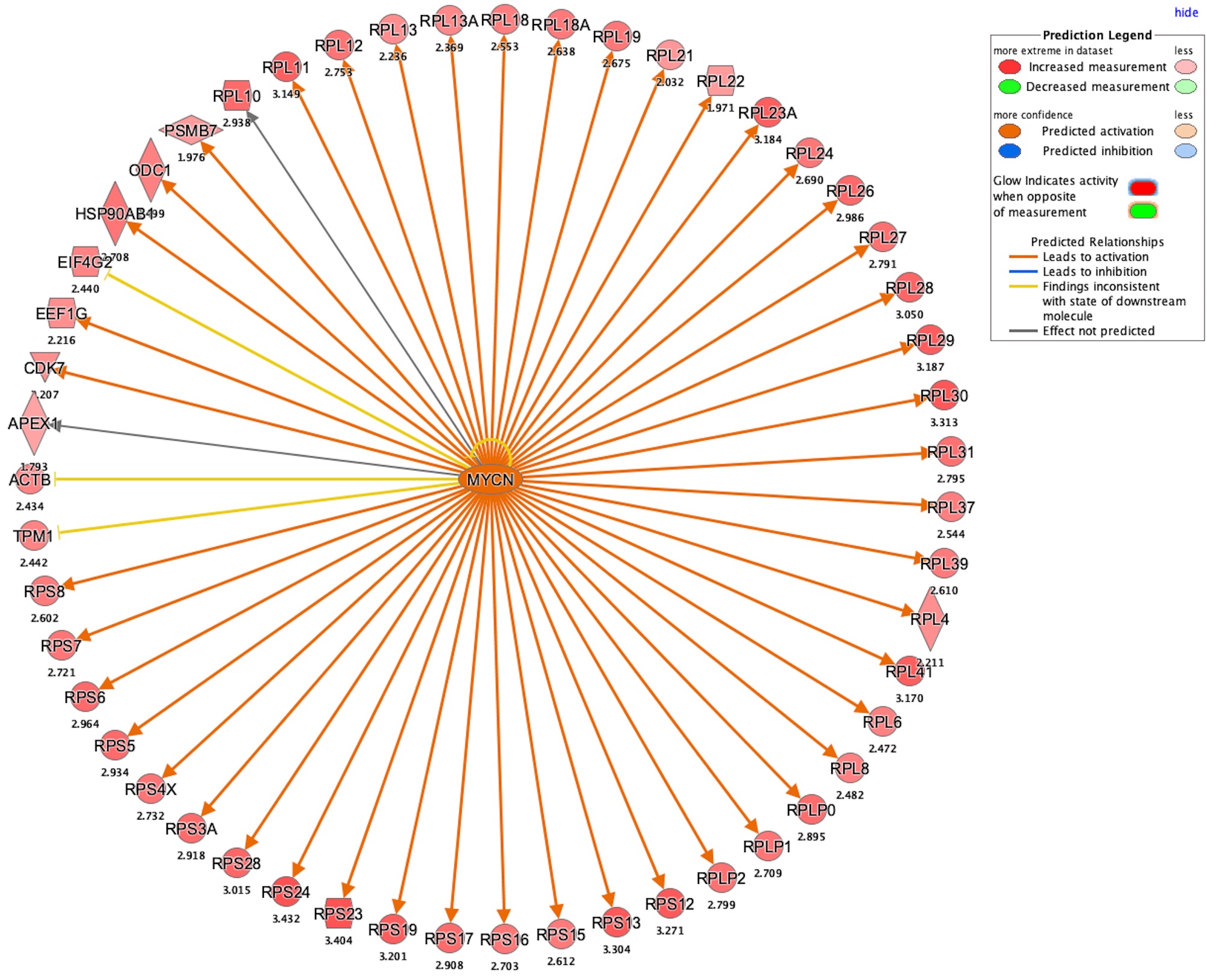


Supplementary Figure 3: MYCN targets. Targets of the upstream regulator MYCN that are among the 397 highly concordant and high-confident orthologous genes between human and zebrafish oocytes. Numbers below the target genes show log10 of the average transcripts per million (TPM) values. Intensity of the red color is proportional to the degree of up-regulation (i.e. high TPM).


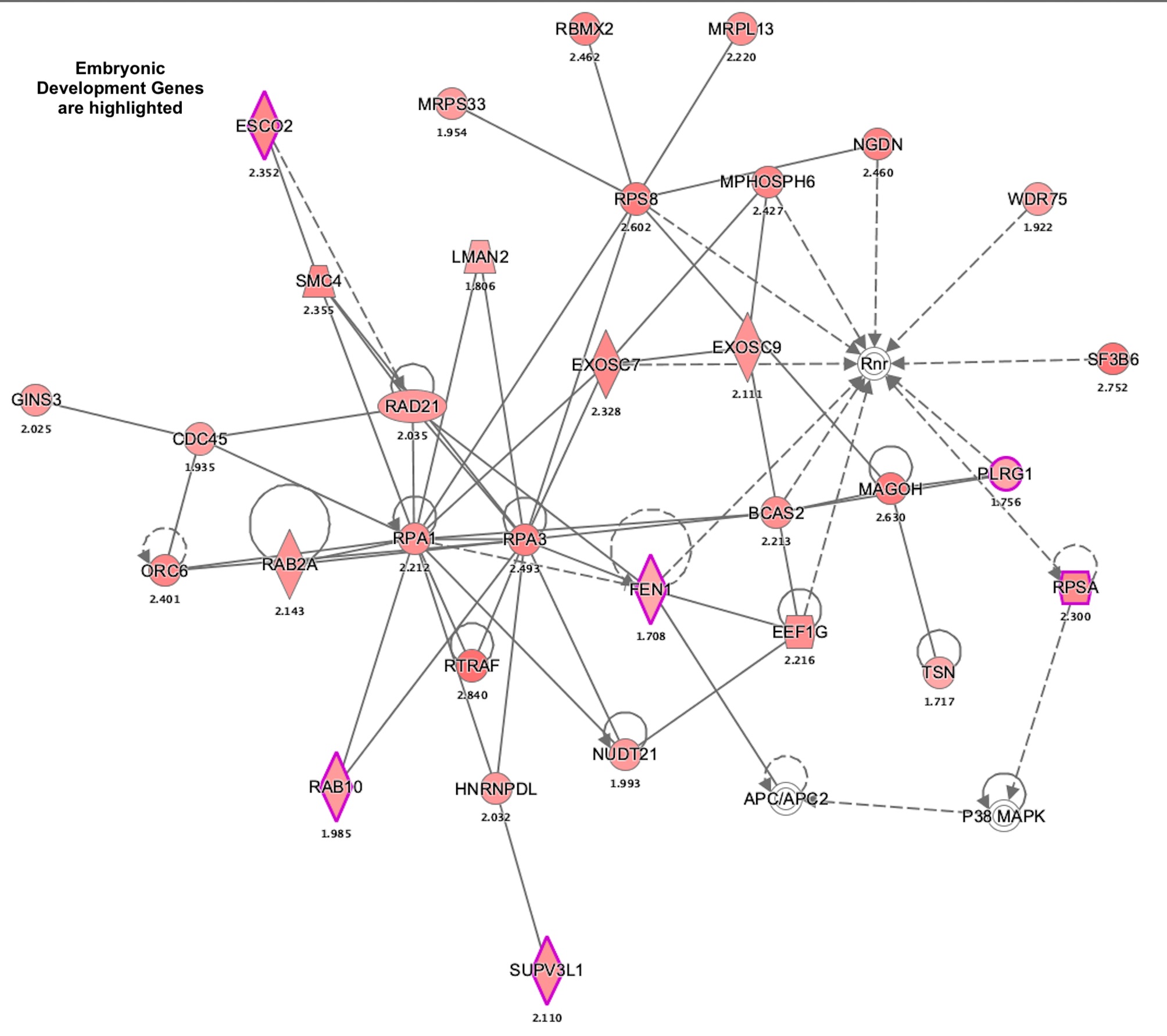


Supplementary Figure 4: Gene interaction network. Interaction network among a subset of the 397 highly concordant and high-confident orthologous genes between human and zebrafish oocytes. Numbers below the target genes show log10 of the average transcripts per million (TPM) values. Intensity of the red color is proportional to the degree of up-regulation (i.e. high TPM). Genes that belong to the “Embryonic Development” functional category are highlighted with a pink outline.


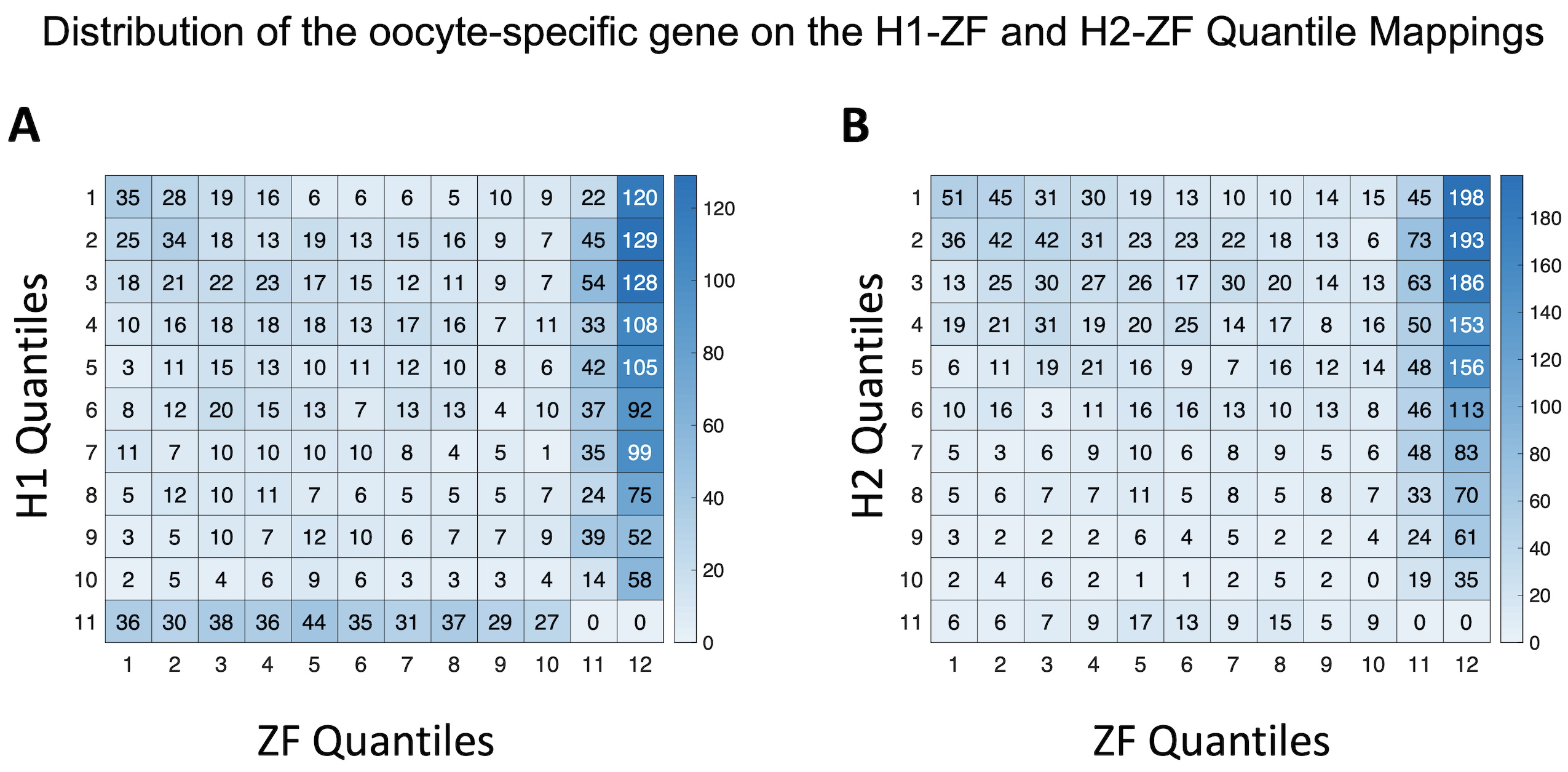


Supplementary Figure 5: Identification of the 3,493 oocyte-specific genes on the quantile mapping described in Figures 2A,B. (A) H1 vs. ZF, (B) H2 vs. ZF. Column 11: oocyte-specific genes that are expressed in human, have a high-confident orthologue in zebrafish, but are not expressed in zebrafish; Column 12: oocyte-specific genes that are expressed in human but do not have a high-confidence orthologue in zebrafish. Row 11: oocyte-specific genes that are expressed in zebrafish, have a high-confident orthologue in human, but are not expressed in human.
